# Supplementary material for: The diagnostic value of native kidney biopsy in low grade, subnephrotic, and nephrotic range proteinuria: A retrospective cohort study
Source: PLoS One. 2022 Sep 2;17(9):e0273671. doi: 10.1371/journal.pone.0273671 (PMC9439248; doi:10.1371/journal.pone.0273671)
Supplement: S1 Table — (DOCX) [file pone.0273671.s001.docx]

**Suppl. Table 1: Frequencies of histological diagnoses by proteinuria-defined subgroup**

| **Biopsy diagnosis** | **Group A**  **UPCR <300mg/g creatinine**  **n=106** | **Group B**  **UPCR 300-3500 mg/g creatinine**  **n=305** | **Group C**  **UPCR >3500 mg/g creatinine**  **n=162** |
| --- | --- | --- | --- |
| **Diabetic nephropathy** | 5 (4.7%) | 15 (4.9%) | 23 (14.1%) |
| **Hypertensive nephropathy** | 13 (12.1%) | 27 (8.8%) | 9 (5.5%) |
| **MCGN** | 1 (0.9%) | 5 (1.6%) | 19 (11.7%) |
| **IgA nephropathy** | 23 (21.5%) | 72 (23.5%) | 22 (13.5%) |
| **FSGS** | 1 (0.9%) | 10 (3.3%) | 14 (8.6%) |
| **Interstitial nephritis** | 8 (7.5%) | 19 (6.2%) | 4 (2.5%) |
| **Lupus nephritis** | 3 (2.8%) | 17 (5.6%) | 11 (6.7%) |
| **ANCA as. GN** | 4 (3.7%) | 48 (15.7%) | 6 (3.7%) |
| **TMA** | 4 (3.7%) | 16 (5.2%) | 7 (4.3%) |
| **ATN** | 11 (10.3%) | 27 (8.8%) | 2 (1.2%) |
| **Membranous nephropathy** | 2 (1.9%) | 13 (4.2%) | 25 (15.3%) |
| **COL4-NP** | 13 (12.3%) | 5 (1.6%) | 0 (0.0%) |
| **Amyloidosis** | 1 (0.9%) | 2 (0.7%) | 11 (6.7%) |
| **Plasma cell dyscrasia** | 4 (3.7%) | 5 (1.6%) | 3 (1.8%) |
| **Other** | 13 (12.1%) | 24 (7.8%) | 6 (3.7%) |

Data presented as n (%); *MCGN* minimal change glomerulonephritis, *IgA nephritis* Immunoglobulin A nephritis, *FSGS* fokal segmental glomerulonephritis, *ANCA as. GN* Antineutrophil Cytoplasmic Antibodies associated glomerulonephritis, *TMA* Thrombotic Microangiopathy, *ATN* acute tubular necrosis, *COL4-NP* Collagen type 4 associated nephropathy*.*
