# Supplementary material for: The diagnostic value of native kidney biopsy in low grade, subnephrotic, and nephrotic range proteinuria: A retrospective cohort study
Source: PLoS One. 2022 Sep 2;17(9):e0273671. doi: 10.1371/journal.pone.0273671 (PMC9439248; doi:10.1371/journal.pone.0273671)
Supplement: S2 Table — (DOCX) [file pone.0273671.s002.docx]

**Suppl. Tab. 2: Biopsy complication**

| ***Complication*** | ***Total*** | ***Group A***  ***<300mg/g creatinine*** | ***Group B***  ***300-3500mg/g creatinine*** | ***Group C***  ***>3500mg/g creatinine*** |
| --- | --- | --- | --- | --- |
| No | 507 (88.5) | 92 (86.8%) | 274 (89.9%) | 141 (87.6%) |
| Minor | 53 (9.3%) | 13 (12.3%) | 25 (8.2%) | 15 (9.3%) |
| Major | 12 (2.1%) | 1 (0.9%) | 6 (2.0%) | 5 (3.1%) |

Data shown n (%)

Minor complications: hematoma/Av-fistula in post-biopsy sonography without the need of therapeutic intervention or post-biopsy cutaneous infection. Major complications: hospitalization according post-biopsy hematoma, AV-fistula or infection, Requirement of blood transfusion, intensive care surveillance or patient death.
