# Supplementary material for: The diagnostic value of native kidney biopsy in low grade, subnephrotic, and nephrotic range proteinuria: A retrospective cohort study
Source: PLoS One. 2022 Sep 2;17(9):e0273671. doi: 10.1371/journal.pone.0273671 (PMC9439248; doi:10.1371/journal.pone.0273671)
Supplement: S3 Table — (DOCX) [file pone.0273671.s003.docx]

**Suppl. Tab. 3 Specific causes of mortality**

| ***Specific causes of death*** | ***Group A  n=5*** | ***Group B***  ***n=18*** | ***Group C***  ***N=21*** |
| --- | --- | --- | --- |
| ***Bacterial infection/sepsis*** | 2 (40%) | 8 (44.4%) | 8 (38.1%) |
| ***Acute respiratory distress syndrome (ARDS)*** | 1 (20%) | - | 1 (4.8%) |
| ***Active malignancy*** | - | 2 (11.1%) | 2 (9.5%) |
| ***Gastrointestinal perforation/bleeding*** | - | 2 (11.1%) | - |
| ***Cardiovascular disease*** | - | 2 (11.1%) | 5 (23.8%) |
| ***Acute on chronic liver failure  (ACLF)*** | - | 3 (16.7%) | 2 (9.5%) |
| ***Acute kidney injury*** | 1 (20%) | 1 (5.6%) | - |
| ***Pulmonary disease*** | 1 (20%) | - | 1 (4.8%) |
| ***Unknown*** | - | - | 2 (9.5%) |

Data shown n (%)
